# Murine Models for *Trypanosoma brucei gambiense* Disease Progression—From Silent to Chronic Infections and Early Brain Tropism

**DOI:** 10.1371/journal.pntd.0000509

**Published:** 2009-09-01

**Authors:** Christiane Giroud, Florence Ottones, Virginie Coustou, Denis Dacheux, Nicolas Biteau, Benjamin Miezan, Nick Van Reet, Mark Carrington, Felix Doua, Théo Baltz

**Affiliations:** 1 UMR 5234, Centre National de Recherche Scientifique, IFR66, Université Bordeaux 2, Bordeaux, France; 2 Projet de recherches cliniques sur la trypanosomiase (PRCT), Daloa, Ivory Coast; 3 Institute of Tropical Medicine Antwerp, Department of Parasitology, Antwerp, Belgium; 4 Department of Biochemistry, University of Cambridge, Cambridge, United Kingdom; New York University School of Medicine, United States of America

## Abstract

**Background:**

Human African trypanosomiasis (HAT) caused by *Trypanosoma brucei gambiense* remains highly prevalent in west and central Africa and is lethal if left untreated. The major problem is that the disease often evolves toward chronic or asymptomatic forms with low and fluctuating parasitaemia producing apparently aparasitaemic serological suspects who remain untreated because of the toxicity of the chemotherapy. Whether the different types of infections are due to host or parasite factors has been difficult to address, since *T. b. gambiense* isolated from patients is often not infectious in rodents thus limiting the variety of isolates.

**Methodology/Principal findings:**

*T. b. gambiense* parasites were outgrown directly from the cerebrospinal fluid of infected patients by *in vitro* culture and analyzed for their molecular polymorphisms. Experimental murine infections showed that these isolates could be clustered into three groups with different characteristics regarding their *in vivo* infection properties, immune response and capacity for brain invasion. The first isolate induced a classical chronic infection with a fluctuating blood parasitaemia, an invasion of the central nervous system (CNS), a trypanosome specific-antibody response and death of the animals within 6–8 months. The second group induced a sub-chronic infection resulting in a single wave of parasitaemia after infection, followed by a low parasitaemia with no parasites detected by microscope observations of blood but detected by PCR, and the presence of a specific antibody response. The third isolate induced a silent infection characterised by the absence of microscopically detectable parasites throughout, but infection was detectable by PCR during the whole course of infection. Additionally, specific antibodies were barely detectable when mice were infected with a low number of this group of parasites. In both sub-chronic and chronic infections, most of the mice survived more than one year without major clinical symptoms despite an early dissemination and growth of the parasites in different organs including the CNS, as demonstrated by bioluminescent imaging.

**Conclusions/Significance:**

Whereas trypanosome characterisation assigned all these isolates to the homogeneous Group I of *T. b. gambiense*, they clearly induce very different infections in mice thus mimicking the broad clinical diversity observed in HAT due to *T. b. gambiense*. Therefore, these murine models will be very useful for the understanding of different aspects of the physiopathology of HAT and for the development of new diagnostic tools and drugs.

## Author Summary

*Trypanosoma brucei gambiense* is responsible for more than 90% of reported cases of human African trypanosomosis (HAT). Infection can last for months or even years without major signs or symptoms of infection, but if left untreated, sleeping sickness is always fatal. In the present study, different *T. b. gambiense* field isolates from the cerebrospinal fluid of patients with HAT were adapted to growth *in vitro*. These isolates belong to the homogeneous Group 1 of *T. b. gambiense,* which is known to induce a chronic infection in humans. In spite of this, these isolates induced infections ranging from chronic to silent in mice, with variations in parasitaemia, mouse lifespan, their ability to invade the CNS and to elicit specific immune responses. In addition, during infection, an unexpected early tropism for the brain as well as the spleen and lungs was observed using bioluminescence analysis. The murine models presented in this work provide new insights into our understanding of HAT and allow further studies of parasite tropism during infection, which will be very useful for the treatment and the diagnosis of the disease.

## Introduction

Human African Trypanosomiasis (HAT), also called sleeping sickness, is a widespread fatal disease in many rural areas of sub-Saharan Africa caused by protozoan parasites of the genus *Trypanosoma* transmitted by tsetse flies. *Trypanosoma brucei rhodesiense* is responsible for the acute form in East Africa and *Trypanosoma brucei gambiense* induces a chronic form in West and Central Africa [1]–[3]. Without treatment, death occurs from either massive parasitaemia or severe neuropathogenesis. Accurate evaluation of the disease stage in the early haemolymphatic stage or the late encephalitic stage is critical as the treatment for both stages is different. Late stage is often treated with melarsoprol, which induces a fatal reactive encephalopathy in 5% of the cases. There is no current consensus on the diagnostic criteria for CNS involvement and the specific indications for second stage treatments might differ [4]–[6]. Diagnosis relies on the Card Agglutination Test for Trypanosomiasis (CATT) based on the detection of host antibodies directed against conserved major Variable Surface Glycoproteins (VSG) of the parasite coat [7],[8] and on the direct microscopic detection of parasites in blood, lymph nodes or cerebrospinal fluid (CSF). However *T. b. gambiense* infections in humans are known for their low parasitaemia and current diagnostics are prone to false negative results. PCR using specific DNA probes [9]–[12] and loop-mediated isothermal amplification (LAMP) [13],[14] were introduced for the detection of the parasites in infected humans or animals providing better sensitivity and specificity compared to parasitological methods [15]. However experiments performed in this study and by other groups proved that even PCR methods may be limited in the case of very low parasitaemia. Thus, new molecular and/or serological methods, possibly based on invariant targets are needed in field diagnosis. Importantly, experimental models for *T. b. gambiense* are limited mainly to subacute or chronic infections since only a few *T. b. gambiense* isolates could be propagated in rodents such as mice [16],[17], cyclophosphamide-immunosuppressed mice [18], severely immunodeficient mice [19] or particular rodent species, *Mastomys natalensis* [20]. Therefore, the variety of *T. b. gambiense* isolates that might be characterised and studied for their *in vivo* behavior is limited. In this study, we have isolated a range of different *T. b. gambiense* stocks from HAT patients and adapted them to *in vitro* culture conditions. These isolates were characterised by molecular fingerprinting and tested for mice infectivity. As they induced different types of infections in BALB/c mice, ranging from chronic to silent infections, the presence and the localization of the parasites, as well as the immune responses of infected mice were addressed.

## Materials and Methods

### Isolation and *in vitro* culture of parasites

CSF samples were obtained from passively or actively HAT detected patients admitted at the Projet de Recherches Cliniques sur la Trypanosomiase (PRCT), Daloa Ivory Coast in 1991. As a reference center from the Ministry of Health, this institution deals with clinical management and research of HAT. Written informed consent was obtained from all patients in this study. The Comite de Protection des Personnes Sud-Ouest et Outre Mer III (cpp.soom3@u-bordeaux2.fr) decided that these procedures did not need approval. Trypanosomes were isolated from CSF and cultured either by *in vitro* culture or by intraperitoneal (i.p.) inoculation in BALB/c mice as described in results. Occasionally, parasites were separated from mice blood cells by anion-exchange chromatography on DEAE-cellulose (DE-52, Whatman Biochemical) [21].

### Mice infection and parasitemia estimation

BALB/c J mice were purchased from Elevage Janvier (5394 Le Genest-St-Isle-France), NOG (NOD.Cg-Prkdescid Il2rgtm1 Wjl/Szj) and NOD/SCID mice (NOD.CB17/ICRT-Prkdescid/J) were bred locally in specific pathogen-free conditions and used for experiments at five to six weeks of age. All animal studies adhered to protocols approved by the University of Bordeaux 2 animal care and use committee and the Commission de Genie Genetique (Direction Generale de la Recherche et de l'Innovation). Groups of 4–6 mice were infected i.p. with either a high load (1–5×10^6^) or a low load (10^3^) of *in vitro* or *in vivo* (NOD/SCID mice) expanded parasites in a 250 µl final volume of PBS (phosphate-buffered saline : 137 mM NaCl, 10 mM Phosphate, 2.7 mM KCl, pH 7.4) containing 1% glucose. Trypanosomes were also inoculated in BALB/c mice immunosuppressed 24 h before infection with cyclophosphamide (200 mg/kg of bodyweight; Sigma-Aldrich, Saint-Quentin Fallavier, France). Mice tail-blood was collected onto a slide and parasitaemia was determined microscopically based on the observation of at least 200 fields at a 400 magnification. The limit of detection was estimated at 10^4^ parasites/ml. To convert the actual concentration of parasites in the blood, a conversion table was made by diluting known numbers of parasites in mouse blood. When parasitaemia was higher than 10^6^ parasites/ml, parasites were directly counted in a haemacytometer by diluting the tail-blood in PBS.

### Parasite DNA and total mRNA purification

Genomic DNA was isolated from the trypanosomes by standard methods [22] and stored at 4°C until needed. Total RNA was extracted from the trypanosomes using RNeasy® mRNA Mini Kit (Qiagen, Hilden, Germany). mRNA was purified from total RNA using Oligotex® mRNA Minikit (Qiagen). The extracted mRNA was kept frozen at −80°C until needed. For PCR detection, a 200 µl sample of whole blood was collected in heparinized capillaries and DNA was extracted with the QIAamp DNA mini kit (Qiagen) according to the manufacturer's instructions and stored at −20°C.

### VSG analysis

To amplify only VSG-encoding transcripts, RT-PCR amplification was performed using a forward primer designed to a conserved nucleotide sequence of the mini-exon found at the 5′ end of trypanosome mRNAs and a reverse primer designed to a conserved nucleotide sequence found at the 3′ untranslated region of VSG mRNAs of trypanosomes belonging to the Trypanozoon subgenus. The forward primer used was IL07725 designed to contain a KpnI site (5′-CGG GTA CCT AGA ACA GTT TCT GTA CTA TAT TG-3′) and the reverse primer was IL07722 designed to contain a BamHI site (5′-CGG GAT CCA GGT GTT AAA ATA TA-3′) [23]. The reaction was carried out using Invitrogen One-Step RT-PCR kit according to the protocol provided by the supplier. Briefly, a 50 µl reaction mixture was prepared containing 0.1 µg mRNA, 0.2 µM of each primer, and 25 µl Reaction Mix 2× containing dNTP, RT-PCR Enzyme and Buffer. RT-PCR amplification was performed as follow: 45°C for 30 min, 94°C for 2 min, 5 touch-down cycles (94 °C for 15 sec, from 55°C to 50°C for 30 sec, 70°C for 2 min) and 34 cycles (94°C for 15 sec, 50°C for 30 sec and 70°C for 2 min) and finally 70°C for 10 min. RT-PCR products were purified on Sephacryl® S-300 column (GE Healthcare Life Sciences, Orsay, France) and cloned into the pTopo vector (Invitrogen, Cergy Pontoise, France). The plasmids were used to transform *Escherichia coli* XL1 blue, which were subsequently plated on selective media for the isolation of recombinants. Plasmids were purified from individual bacterial colonies using the Wizard® Plus SV Minipreps DNA Purification System (Promega, Charbonnières, France). The size of inserts was determined by PCR using vector primers and at least 10 clones of each isolate were sequenced. Similarity searches of GenBank/NCBI database were performed with the program BLAST (http://www.ncbi.nlm.nih.gov/BLAST/) using the default matrix. Alignment of two sequences was performed using the program FASTA (http://fasta.bioch.virginia.edu/fasta_www/cgi/search_frm2.cgi).

### Microsatellites and minisatellites analysis

Microsatellite analysis of the MORF2-CA, M6C8-CA, MT3033-AC/TC and MEST19-AT/GT loci was performed as previously described [24] except that the PCR products were analysed on both an ABI 377 DNA and an ABI 3130 XL sequencer (PE, Applied Biosystems). PCR and analysis of the PARP minisatellite locus (PE procyclin repetition) [25] was performed as described for the microsatellites using PARP-S (GACGATACCAATGGCACTG) and PARP-AS-6-FAM© (TGCGAACGGAAGTGCAAC) primers.

### PCR detection of parasites

In order to avoid loss of sensitivity due to blood contamination with polymerase enzyme inhibitors [26], commercial columns for blood DNA extraction ensuring reproducibility and a high degree of purification were used for all samples (QIAamp DNA mini kit from QIAGEN) [27]. As PCR detection based on a single target is often doubtful for very low parasitaemia, three different targets were used to validate the presence of trypanosome DNA in the blood of infected mice: 1) KIN primer set (Kin1: 5′-GCGTTCAAAGATTGGGCAAT-3′; Kin2: 5′-CGCCCGAAAGTTCACC-3′) designed by McLaughlin *et al.* [28] amplifying a 360 bp product from a highly conserved and highly represented region (100–200 copies per parasite) named ITS1 and located between 18S and 5.8S rRNA genes, 2) TBR primer set (TBR1: 5′-CGAATATTAAACAATGCGCAG-3′; TBR2: 5′-AGAACCATTTATTAGCTTTGTTGC-3′) [29] shown to be highly sensitive for HAT diagnosis [26],[30] and targeting a 177 bp repeated satellite DNA; and 3) the primers designed by Deborggraeve *et al.*, [27] and frequently used in diagnosis test for HAT as they amplify a 106 bp sequence in conserved 18S rRNA (18S-F: 5′-CGCCAAGCTAATACATGAACCAA-3′; 18S-R: 5′-TAATTTCATTCATTCGCTGGACG-3′). All PCR were carried out in a final volume of 25 µl containing 0.8 µM of each primer, 0.2 mM of each deoxyribonucleotide, 1× incubation buffer with 2.5 mM MgCl_2_, 1 unit of HotStar Taq polymerase (QIAGEN, Hilden, Germany) and 5 µl of extracted DNA. PCR conditions with Kin, TBR and 18S primers were performed according to the method previously described [27],[31],[32], respectively. Kin1-2 primers anneal in the conserved regions of the 18S and 5.8S rRNA genes to amplify the ITS1. TBR1-2 primers are specific for *Trypanosoma brucei sensu lato* [33]. 18S primers amplify a sequence of the *Trypanosomatidae* 18S rRNA gene. Briefly, PCR conditions with Kin primers were as follow: an initial step of 15 min at 94°C to activate the HotStar *Taq* polymerase; four cycles of amplification with 1 min denaturation at 94°C, 1 min hybridization at 58°C and 1 min elongation steps at 72°C; eight cycles of amplification with 1 min denaturation at 94°C, 1 min hybridization at 56°C and 1 min elongation steps at 72°C; 23 cycles of amplification with 1 min denaturation at 94°C, 1 min hybridization at 54°C and 1 min elongation steps at 72°C; and a final extension step of 5 min at 72°C [31]. The amplification conditions with TBR were an initial denaturation at 94°C for 15 min, 45 cycles of 94°C for 1 min, 56°C for 1 min and 72°C for 1 min and final extension was at 72°C for 5 min [32]. PCR conditions with 18S primers were an initial denaturation step of 94°C for 15 min, 40 cycles of 94°C for 30 s, 60°C for 30 s, and 72°C for 30 s with a final extension at 72°C for 5 min [27]. PCR amplification was performed in triplicate in three different assays. Purified *T. b .gambiense* DNA was used as positive control and a negative control without DNA was performed. 10 µl of reaction samples or controls were tested on 1.5% agarose gel, stained with 0.5 µg/ml ethidium bromide. Results were positive when specific size products were observed.

### IgM quantification

Sandwich ELISA was used to quantify and to determine the total IgM level in mice. Goat anti-mouse μ-chain antibodies as a capture antibody (Jackson ImmunoResearch, Baltimore) were coated in a ninety-six-well microplate (Nalge Nunc International) with 0.2 µg/well at 4°C overnight. After washing in PBS containing 0.05% tween-20 (washing buffer) three times, the plate was incubated with 100 µl/well of 3% BSA-PBS for 30 min at 37°C. The wells were then reacted with test samples which were diluted in 1% BSA-PBS (100 µl/well) for 2 hours at 37°C followed by reaction with 100 µl/well of goat anti-mouse IgM antibodies conjugated with horseradish peroxidase (Southern Biotech Birmingham, USA) diluted 1∶8000 in PBS-Tween buffer for 1.5 hours at 37°C. The plate was washed in washing buffer three times after each reaction. The development was performed by using ABTS, 2,2′-azino-di-(3-ethylbenzthiazoline sulfonic acid) (Sigma-Aldrich Corp, Saint-Quentin Fallavier, France) and was stopped by addition of 10 mM NaOH, 1 mM EDTA (100 µl/well). The absorbance at 405 nm was measured. The level of mouse IgM was normalized by mouse reference serum (Gentaur Belgium).

### Cloning, expression and purification of recombinant proteins

The full length sequence for TgsGP was cloned into the pET-28c(+) vector (Novagen) using specific primers (F: 5′-GCTATTCCATGGGGATGTGGCAATTACTAGCAATAG-3′ containing NcoI site and R: 5′-CCGGAATTCTTAATGGTGATGGTGATGGTTGCTGTGGTGTTTGCCACTTC-3′ containing EcoRI site), expressed in *E. coli* BL21 bacteria according to the manufacturer's instructions and purified in the presence of 8 M urea by immobilized metal ion Ni-affinity chromatography (His-Trap, GE Healthcare). The full-length calflagin gene (Tbg17-19576 corresponding to the 26 kDa protein isoform) was amplified using primers containing KpnI and HindIII sites (F: 5′-TCTAGAGGTACCAATGGGTTGCTCAGGATCCAAGAAC-3′ and R: 5′-CTGCAGAAGCTTC TAGGCAGAACCCTCGGCCGCAGG-3′) and the insert was cloned into the pMalE vector (New England Biolabs). After expression in *E. coli* BL21 bacteria, the soluble fusion protein was purified by maltose-affinity chromatography according to the manufacturer's instructions. The extracellular domains of ISG64, ISG65 and ISG75 were amplified from genomic DNA and cloned into pET21d. The ISG proteins were expressed in *E. coli* BL21 (DE3) *trxB* bacteria as inclusion bodies. After purification of the inclusion bodies the proteins were solubilized in 6 M guanidinium hydrochloride and purified by Ni-affinity chromatography, refolded by overnight dialysis in 20 mM Tris buffer (pH 8.0) and concentrated by Q-sepharose chromatography. Denatured PFR protein was kindly provided by D. Robinson. The soluble recombinant antigens ISGs and calflagin were used to coat ELISA plates (0.1 µg/well). The antibody response to these antigens was measured in ELISA as previously described [34] by using a 1∶100 dilution of the infected mice sera.

### SDS-PAGE, immunoblotting and recombinant protein strip test

Total protein preparations of bloodstream form trypanosomes were obtained by lysis of live parasites with 2% (wt/v) SDS and heating at 100°C in the presence of a protease inhibitor cocktail (complete Mini, EDTA-free; Roche Diagnostics, GmbH). *T. b. brucei* trypanosome fractions were obtained as described previously [35]. Briefly, trypanosomes (about 10^10^ cells) were washed in phosphate saline glucose (pH 8.0) at 4°C, centrifuged and hypotonically lysed in 15 ml of 5 mM sodium phosphate, pH 8.0 in the presence of a protease inhibitor cocktail. After centrifugation (44,000 g for 8 minutes), the soluble proteins and the cytoskeleton/membrane proteins contained in the pellet (washed four times in lysis buffer and resuspended in an equal volume of 100 mM Tris pH 8.0) were boiled in SDS-PAGE loading buffer. Protein preparations of 10^7^ parasites (from total lysate or each purified fraction) were loaded per well and separated by SDS-PAGE (12%) before transfer onto polyvinyl difluoride (PVDF) membranes (Immobilon-P; Millipore) and processed for Western blotting as described previously [34]. Trypanosome specific antibodies were tested by incubating the blots with infected mice sera (diluted 1∶100). For identification of potential immunoreactive proteins, blots were incubated with dilutions of invariant specific antibodies: mouse anti-PFR2 monoclonal antibody (1∶500), rabbit anti-IS64, ISG65 or ISG75 polyclonal antibodies (1∶100), or a mouse anti-calflagin monoclonal antibody (culture supernatant diluted ½).

A recombinant protein strip test was developed to analyse the mice infected immune response. Briefly, all recombinant proteins were further purified by electroelution after SDS-PAGE and loaded sequentially on a wide PAGE (0.5–2 µg of each protein per strip). ISGs, TgsGP and calflagin were first separated for 15 minutes at 150 V on a 12% SDS-PAGE before loading PFR and finishing the migration at 180 V. After blotting, nitrocellulose membranes were cut into strips and incubated with infected mice sera (diluted 1∶100). After being washed three times in 1 M NaCl, blots were incubated for 1 h with diluted horseradish peroxidase-conjugated anti-rabbit or anti-mouse immunoglobulin G (IgG) (both from Sigma) (1∶10,000). Diaminobenzidine (DAB, Sigma) was used as chromogen.

### Brain immunohistochemistry

Anaesthetized mice were transcardially perfused with 50 ml of ice cold PBS, then with 50 ml of ice cold 2% paraformaldehyde, 0.2% glutaraldehyde in 0.1 M phosphate buffer, pH 7.4. The brains were removed, post-fixed in fresh 2% paraformaldehyde for 5–6 hours at 4°C and cryoprotected overnight at 4°C in a 15% sucrose 0.1 M PO4 buffer, pH 7.4. The brains were then frozen in isopentane at −50°C on liquid nitrogen and stored at −80°C. Sections of 12 µm were cut in cryostat and stored at −80°C [36]. The slides were thawed and dried at room temperature then fixed again in 4% paraformaldehyde for 5 minutes. The endogenous peroxidase activity was quenched with 3% H_2_O_2_ in PBS for 5 minutes. The slides were washed in water then in PBS. The sections were permeabilized and saturated with blocking buffer (1% BSA 0.3% Triton X-100 in PBS) for 30 minutes. The sections were then incubated overnight at room temperature with antibodies (used at a 1∶10000 dilution in blocking buffer) obtained from rabbits that were hyper immunized with a total *T. b. equiperdum* protein extract. The slides were washed in PBS and incubated with a secondary biotinylated anti-rabbit antibody (1∶200) in blocking buffer for 90 min at room temperature. Thereafter the sections were incubated with avidin-biotin complex reagent (Vectastain ABC peroxidase, Vector, ABCYS, Paris, France) according to the manufacturer protocol. The immuno-complex was visualized with a DAB kit (Vector). The sections were dehydrated in graded alcohol, mounted with DePeX (SERVA, Paris, France), and analyzed for the presence of trypanosomes using a Zeiss light microscope. Control sections (without primary antibodies) showed no specific immunostaining.

### Transformation of *T. b. gambiense* with the Rluc-pHD309 plasmid

The Rluc-pHD309 plasmid designed for the integration of the *Renilla* luciferase gene into the β-tubulin region of *T. brucei* Lister 427 bloodstream forms was kindly provided by F. Claes [37] . The Amaxa Nucleofector® system (Lonza, Levallois-Perret, France) was used. It is based upon a combination of free-set programs and “cell-type specific” solutions of unknown composition, which was described for giving vastly improved transfection efficiencies for the protozoan parasite *Plasmodium*. A pellet of 10^7^ parasites was resuspended in 100 µl of Basic Parasite Nucleofector® solution 2, mixed with 15 µg of NotI linearized LucR-pHD309 plasmid and subjected to nucleofection with program X-001. Stably transfected trypanosomes were first cultured 24 h in supplemented IMDM medium (Gibco) as described earlier [37] before selection by adding increasing concentrations of hygromycin (starting and final concentrations: 0.5 µg–3 µg/ml). Single clones, generated by limiting dilution, were expanded and assessed by luciferase activity quantification.

### *In vitro* bioluminescence measuring

The *Renilla* Luciferase Assay System (Promega) was used to measure *in vitro* luciferase activity as described by the manufacturer. Non-transformed *Tgb1135* and Rluc-pHD309-transfected clones were grown up to a total of 1–5×10^6^ parasites and centrifuged at 1500 g for 10 min. The pellet was washed with PBS, resuspended in 20 µl lysis buffer included in the Renilla luciferase assay system (Promega) and subsequently added to 100 µl of the reaction mix. *Renilla* luciferase activity was monitored over 5 min every 10 sec after substrate addition by using an Optima microplate reader (BMG Labtech, Germany) and expressed as relative light units (RLU) per µg protein.

### *In vivo* and *ex vivo* real time bioluminescence imaging

At different time intervals after infection, mice were anaesthetized with isofluorane and injected intravenously (retro-orbital i.v.) or intraperitoneally (i.p.) with 20 µg of coelenterazine (Promega) dissolved at 2 µg/µl in ethanol and subsequently 10 µl were diluted in 90 µl of PBS. Light emission was recorded in real time with a Biospace Imaging System (BIOSPACE lab, Paris, France). Measurements started immediately after i.v. or i.p. substrate injection and the results correspond to the values measured when the signal is optimal and stable for at least 3 min. The signal was integrated for 100 sec and values are expressed as p/s/sr (photons/second/steradian) (maximum and minimum values are fixed respectively at 100 and 10, smoothing = 2.5). For *ex vivo* bioluminescence imaging, mice were sacrificed and organs removed and soaked for 5 min in coelenterazine (20 µg/ml PBS) before recording signals as described before. Quantification of BLI signals was performed on selected regions of interest (ROI). For the comparison of signals from the same organ between different mice, the signal was integrated for 100 sec, the area of the ROI was kept constant and the intensity was given as p/s/cm^2^/sr after subtracting the background photon emission values obtained for each organ from non infected mice.

## Results

### Isolation and culture adaptation of *T. b. gambiense* field isolates

Isolation and growth of *T. b. gambiense* from patients remains difficult and is the limiting step in analysis. Here, we have adapted an *in vitro* culture system based on fibroblast feeder cells [38],[39]. CSF (6–8 ml) was collected aseptically by lumbar puncture from 37 patients who were already in the second stage of HAT. After centrifugation, half of the sample was inoculated intraperitoneally (i.p.) into BALB/c mice for *in vivo* amplification. The other half was transferred to culture medium in 24-well plates containing a confluent feeder layer of *Microtus montanus* embryonic fibroblasts. The culture medium was MEM medium [40] supplemented with 3.5% fetal calf serum and 15.5% horse serum instead of goat serum. Kanamycin (400 µg/ml) and nystatin (400 U/ml), were added to avoid contamination. The plates were incubated at 37°C in a 4% CO_2_/96% air incubator. These conditions were optimal for *in vitro* culture of low CSF parasitaemia. Following 12 days of culture, 34/37 isolates were successfully amplified in culture whereas only 6/37 were infective in BALB/c mice (blood isolates) and subsequently caused chronic infections.

**Figure 1:**
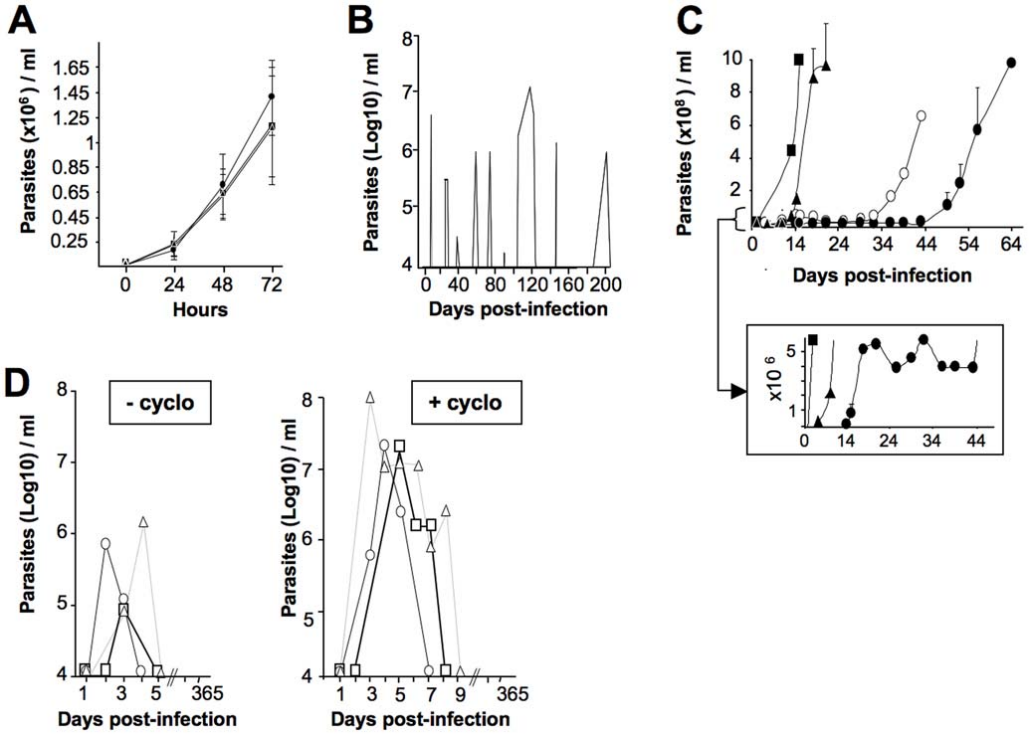
*In vitro* and *in vivo* growth characteristics of *T. b. gambiense* field isolates. A. *In vitro* culture of *Tbg*1122, *Tbg*1166 and *Tbg*1135 isolates after adaptation to axenic culture conditions. Cultures were seeded with adapted trypanosomes (5.10^4^/ml) in supplemented MEM medium and enumerated every 24 h. The mean trypanosome densities±standard error of the mean of 4 independent cultures is presented. Similar doubling times were obtained with the three isolates (15.9 h, 15.8 h and 14.6 h respectively). B–D. Parasitaemia levels in immunocompetent (BALB/c), immunodeficient (NOD/SCID and cyclophosphamide-treated BALB/c) mice infected with the different field isolates. Parasitaemia was measured from tail-blood either by direct observation of the wet films under the microscope or by using a haemacytometer. The limit of detection was estimated at about 10^4^ parasites/ml. B. Represents the results of one representative BALB/c mouse (n = 6) infected i.p. with 10^6^ of the *Tbg*945 blood isolate. All infected mice showed successive waves of parasitaemia and died within 6–8 months PI. C. Represents the mean parasitaemia in NOD/SCID mice infected with 10^3^ *Tbg*1122c (n = 4), *Tbg*1166c (n = 6), *Tbg*1135c (n = 9) or *Tbg*1135b (n = 10) isolates. D. Represents the results of one representative BALB/c mouse infected with 1–5×10^6^ *Tbg*1122b, *Tbg*1166b (n = 10) or *Tbg*1135b blood (n = 6) isolates with (+cyclo) or without (−cyclo) prior administration (24 h before infection) of cyclophosphamide (200 mg/kg). Solid symbols indicate culture isolates, open symbols indicate blood isolates of *Tbg*1166 (▴ , ▵), *Tbg*1122 (▪ , □) and *Tbg*1135 (• , ○).

From these, we focused on three cultured isolates which failed to infect immunocompetent mice: *Tbg*1122c, *Tbg*1166c and *Tbg*1135c (c representing culture isolate) and one derived from a mouse infection, *Tbg*945b (b representing blood isolate). The culture isolates were rapidly adapted to axenic culture conditions, MEM medium supplemented with 10% fetal calf serum, as described previously [40] and cloned for further characterisation. These clones showed a normal *in vitro* expansion with similar growth rates: 15.9, 15.8 and 14.6 hour doubling time for *Tbg*1122c, *Tbg*1166c and *Tbg*1135c respectively (Fig. 1A). Despite numerous attempts, *Tbg*945b could not be adapted to axenic culture conditions.

### Molecular characterisation of *T. b. gambiense* field isolates

*T. b. gambiense* comprises of two different groups: Group 1 is characterised by its low virulence in rodents and a chronic infection in humans, whereas Group 2 is virulent in rodents and humans [41]. Using mini- and micro-satellite analysis [24], we could clearly identify the 4 isolates, reported above, as Group1 *T. b. gambiense* (markers genotype: MORF2,16/16; MEST19,22/22; PE procyclin All1,15), even though polymorphism was observed with other mini- and micro-satellite markers such as MT3033 (30/x) and M6C8 (13/x) (Table 1). Moreover, all four isolates expressed the Group 1 *T. b. gambiense* specific glycoprotein TgsGP mRNA [10],[42] (data not shown). To further characterise these isolates, their expressed VSGs were analyzed (data not shown). The VSG expressed by these clones are very similar (68%–96% identity) to VSG sequences identified in the *T. b. gambiense* and *T. b. brucei* sequence data-base (www.genedb.org).

**Table 1:** Minisatellites and microsatellites analyses.

Identification	Focus	Year	Host	Microsatellites (Repetition number or size)				
				MORF2-CA	M6C8-CA	MT3033-AC/TC	MEST19-TA/CA	PE procyclin repetition number
				All. 1-2	All. 1-2	All. 1-2	All. 1-2	All. 1-2-3-4-5
***Trypanosoma brucei gambiense*** ** Type I**								
27/7	Ivory Coast		Human	16 - 16	13 - 53	30 - 48	22 - 22	15 – 20 – 21 - 24
Zakaria	Ivory Coast		Human	16 - 16	13 - 53	30 - 49	22 - 22	15 – 20 – 21 - 24
LiTat-1/1	Ivory Coast	1952	Human	16 - 16	13 - 44	30 - 43	22 - 22	15 – 19 – 20 - 21
1122	Ivory Coast	1991	Human	16 - 16	13 - 44	30 - 44	22 - 22	15 – 20 - 21
1166	Ivory Coast	1991	Human	16 - 16	13 - 44	30 - 43	22 - 22	15 – 20 - 21
1135	Ivory Coast	1991	Human	16 - 16	13 - 43	30 - 43	22 - 22	15 – 20 - 21
945	Ivory Coast	1991	Human	16 - 16	13 - 43	30 - 30	22 - 22	15 – 20 - 21
***Trypanosoma brucei gambiense*** ** Type II (Bouaflé)**								
HTAG/107-1	Ivory Coast	1986	Human	13 - 48	23 - 37	14 - 14	24 - 25	21 – 22 – 23 – 26 - 28
TH2 (78E)	Ivory Coast	1978	Human	13 - 49	38 - 48	19 - 22	31 - 40	22 – 23 – 26 - 28

The genotype of each isolate (*Tbg*945, *Tbg*1122, *Tbg*1166 and *Tbg*1135) was analyzed as described earlier [24] by determining the number of repeats per allele and compared to group 1 and 2 genotypes.

### Mice infectivity properties of *T. b. gambiense* field isolates

To assess infectivity of these four isolates, different experimental models of mice were used: immunocompetent BALB/c mice, cyclophosphamide-immunosuppressed BALB/c mice and severely immunodeficient NOD/SCID mice. Mice were infected i.p. with either a high load (1–5×10^6^) or a low load (1×10^3^) of parasites and animals were monitored for parasitaemia, paresis and survival. Immunocompetent mice inoculated with either a high or a low load of *Tbg*945b parasites always developed a classical chronic infection with successive waves of parasitaemia (undulating between 1.2×10^6^ and 1.2×10^7^ parasites/ml) and death within 6–8 months post-infection (PI) (Fig. 1B). However, mice inoculated with a high load of *Tbg*1122c or *Tbg*1166c parasites developed a sub-chronic infection characterised by a single wave of parasitaemia (<1×10^5^ parasites/ml) 3–4 days PI followed by the absence of detectable parasites in the blood by direct microscopic observation (limit of parasitaemia detection >10^4^ parasites/ml). In contrast, mice infected with *Tbg*1135c parasites developed a silent infection devoid of any detectable parasite during the 12 months of monitoring (data not shown). Identical results were obtained when mice were infected with a low load (10^3^ parasites) of any of the culture isolates. No parasite could be detected by microscopic observation and most of the mice survived more than 12 months without any clinical signs of disease.

As resistance or susceptibility to trypanosomiasis is associated with non-specific and specific immune responses, we investigated the infectivity of the *T. b. gambiense* field isolates in NOD/SCID mice which are lymphocyte deficient and have reduced NK, complement and macrophage activities. A low dose (1×10^3^ parasites) of chronic *Tbg*945b (not shown) or sub-chronic *Tbg*1122c and *Tbg*1166c induced an acute infection with high parasitaemia (>1×10^9^ parasites/ml) and death of animals within 10–11 or 20 days PI respectively. The silent *Tbg*1135c isolate induced an infection characterised by a low parasitaemia (<10^6^ parasites/ml) lasting 50 to 60 days, followed by relapse with high parasitaemia and death of animals (Fig. 1C). As the relapse might result from a host adaptation, we compared the infectivity of the parasites isolated directly from the blood after relapse (*Tbg*1135b) with the infectivity of the culture isolate (*Tbg*1135c). Infection of NOD/SCID mice with *Tbg*1135b resulted in a relapse and death of the animals within a shorter time (30–42 days PI) compared with *Tbg*1135c (50–70 days). No difference was observed for *Tbg*1122 and *Tbg*1166 culture and blood isolates (data not shown). Furthermore, we characterised the VSG genes expressed by the parasites before infection (*Tbg*1135c) and during the relapse (*Tbg*1135b). Comparison of cDNA sequences encoding VSG showed that *Tbg*1135b and *Tbg*1135c expressed different VSGs (data not shown). These results were confirmed by the absence of cross-reactivity between *Tbg*1135b and *Tbg*1135c by using variant specific anti-sera (data not shown).

In order to assess the influence of the early antibody response, the infectivity of a high load of the different blood isolates (*Tbg*1122b, *Tbg*1166b and *Tbg*1135b) was tested in BALB/c mice with or without prior administration of a high dose of cyclophosphamide (200 mg/kg). Similar parasitaemia profiles, characterised by an early and transient parasitaemia followed by the absence of detectable parasites in the blood by direct microscope observation, were observed in immunocompetent and cyclophosphamide-treated mice (Fig. 1D). Nevertheless, the peak of parasitaemia was higher in intensity and duration in immunosuppressed (10^7^–10^8^ parasites/ml and 5 to 9 days) compared to untreated mice (10^6^ parasites/ml and 2 to 5 days). Moreover, when BALB/c mice were treated with cyclophosphamide before the infection, 20% (7 out of 34 mice) of the infected animals developed a hind leg paresis within 6–10 months PI compared to 6% of the immunocompetent infected mice (6 out of 104 mice infected during the whole study). Parasitaemia of cyclophosphamide-treated mice inoculated with the *Tbg*1135c isolate remained cryptic despite immunosuppression. Furthermore, when mice infected with *Tbg*1122b, *Tbg*1166b, *Tbg*1135b and *Tbg*1135c were treated with cyclophosphamide during their cryptic phase of parasitaemia, no parasite burst could be observed. In mice infected with the chronic isolate *Tbg*945b, however, a rapid relapse of the parasitaemia is always observed (data not shown).

As low parasitaemia escapes detection by direct tail-blood observation, we addressed the presence of parasites in blood by specific PCR. In order to gain better specificity and sensitivity, three different multi-copy genes present in the Trypanozoon subgenus were targeted [27]–[29]. When triplicate PCR gave three positive or three negative results for at least two of the three PCR targets, the sample was considered as + or − respectively. However, for discordant results (with only one positive PCR), the sample was considered as doubtful (±). Parasite PCR detection was positive until 12 months PI in all immunocompetent infected mice irrespective of the field isolate, the parasite load and the amplification of the parasites before inoculation (culture or blood isolates). Nevertheless, the presence of parasites in the blood might fluctuate during infection as some samples were found negative during the 12 months of monitoring. Table 2 illustrates the results obtained with mice infected with 1×10^3^ parasites of the silent *Tbg*1135c isolate.

**Table 2:** PCR detection of Trypanosomes in the blood of BALB/c mice infected with 10^3^ parasites of the silent *Tbg*1135c isolate.

BALB/c mice	Months post-infection		
	2	4	9
**a**	+	+	±
**b**	+	+	+
**c**	±	+	+
**d**	+	±	+
**e**	+	+	+

Detection is based on the amplification of 3 *Trypanosoma brucei* specific gene targets. The + sign represents a positive result (2–3 out of 3 targets amplification). ± represents a doubtful result (1 out of 3 targets amplification).

### Non-specific and specific humoral immune response of BALB/c mice infected with *T. b. gambiense* field isolates

**Figure 2:**
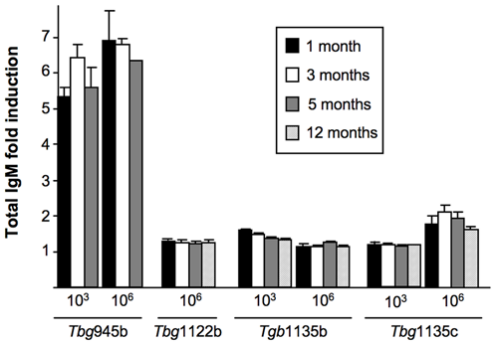
Time course of total serum IgM levels in BALB/c mice infected with different *T. b. gambiense* field isolates. Four mice were infected with either a low (10^3^) or a high (10^6^) load of *Tbg*945b, *Tbg*1122b, *Tbg*1135b or *Tbg*1135c isolates then IgM levels were quantified by ELISA test 1 month (black bars), 3 months (white bars), 5 months (gray bars) and 12 months (hatched bars) after infection. IgM levels are expressed as a multiple (mean±standard error) of the level before infection.

As chronic experimental *T. b. gambiense* infections are characterized by macroglobulinemia [43],[44], we investigated the serum immunoglobulin M (IgM) level in different infection models: mice infected with either 1×10^3^ or 1×10^6^ trypanosomes of the chronic (*Tbg*945b), sub-chronic (*Tbg*1122b, *Tbg*1135b) or silent (*Tbg*1135c) isolates. Whereas *Tbg*945b induced a 5 to 7 fold increase in total IgM level in BALB/c mice compared to non-infected mice, as soon as one month PI, the total IgM level in sub-chronic or silent infections was only slightly (less than 2 fold) increased (Fig. 2).

**Figure 3:**
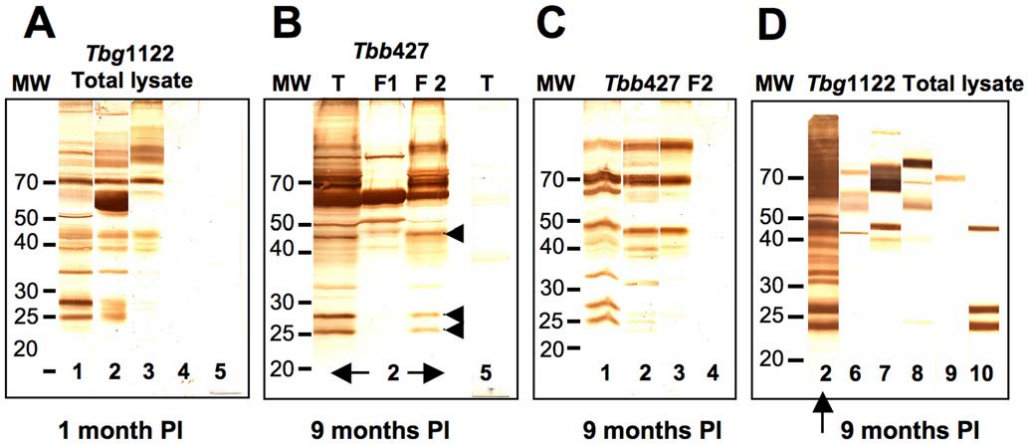
Immunoblotting analysis of the trypanosome antigens recognised during BALB/c mice infections and identification of potential immunoreactive proteins. Different trypanosome protein extracts: (3A,D) *Tbg*1122b total lysate, (3B) *Tbb*427 total lysate (T) and *Tbb*427 fractions containing the soluble proteins (F1) or cytoskeleton/membrane proteins (F2), (3C) *Tbb*427 cytoskeleton/membrane fraction (F2) were subjected to SDS-PAGE and tested by Western blotting against different sera from BALB/c (n = 4) mice infected with (10^3^) parasites, (3A–C): sera from *Tbg*945b (1), *Tbg*1122b (2), *Tbg*1135b (3), *Tbg*1135c (4); non-infected control mice (5) or (3D) antibodies specific for cytoskeleton or membrane proteins: rabbit polyclonal antibodies directed against ISG64 (6), ISG65 (7), ISG75 (8), mouse monoclonal antibodies recognizing PFR2 (9) or calflagin (10). A–C represents the results of one representative immunoblot out of 4 mice tested.

The capacity of trypanosome infections to induce a specific immune response was tested by Western blotting against total parasite extracts subjected to SDS-PAGE (whole lysates of *T. b. gambiense Tbg*1122 or *T. b. brucei Tbb*427). The sera of mice infected with 1×10^3^ parasites of *Tbg*945b or *Tbg*1122b strongly recognised a large panel of proteins with MW ranging from 20 to 100 kDa as soon as one month PI (Fig. 3A) and during the entire time course of infection (identical profiles were observed 9 and 12 months PI, data not shown). The additional specific band strongly recognised by the sera of mice infected with *Tbg*1122 is most likely to correspond to the VSG expressed by *Tbg*1122. The response was less intense with the sub-chronic *Tbg*1135b isolate (1×10^3^ parasites) and no reactivity was observed with the sera of mice infected with the silent *Tbg*1135c isolate (1×10^3^ parasites) with the exception of one serum out of four which recognised a single band migrating at 70 kDa at 9 months PI (data not shown). Since identical Western blot profiles were obtained with whole cell protein extracts of *Tbb*427 (T), the sera were tested against two *Tbb*427 trypanosome lysate fractions (Fig. 3B): a soluble protein fraction (F1) and a cytoskeleton/membrane fraction (F2). As shown in Figure 3B and C, most of the proteins recognised by the sera belong to the cytoskeleton/membrane fraction.

### Characterisation of trypanosome antigens recognised during infection

**Figure 4:**
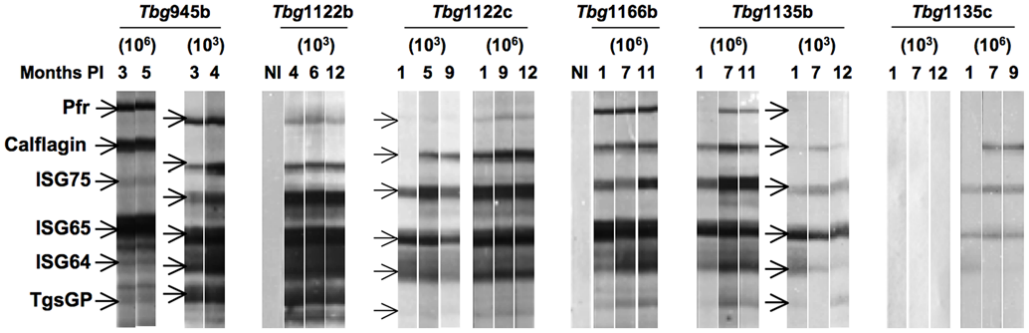
Reactivity patterns of immunoreactive invariant trypanosome proteins during BALB/c mice infections. Four mice were infected with either a low (10^3^) or a high (10^6^) load of *Tbg*945b, *Tbg*1122b, *Tbg*1166b, *Tbg*1135b or *Tbg*1135c isolates and their sera collected at different time points were tested by Western blotting (1/100 dilution) against a strip loaded with recombinant protein: 0.5 µg PFR and ISG75, 1 µg ISG65, ISG64 and TgsGP and 2 µg calflagin. The data are representative of one immunoblot out of 4 mice tested. NI represents the control sera before infection.

**Figure 5:**
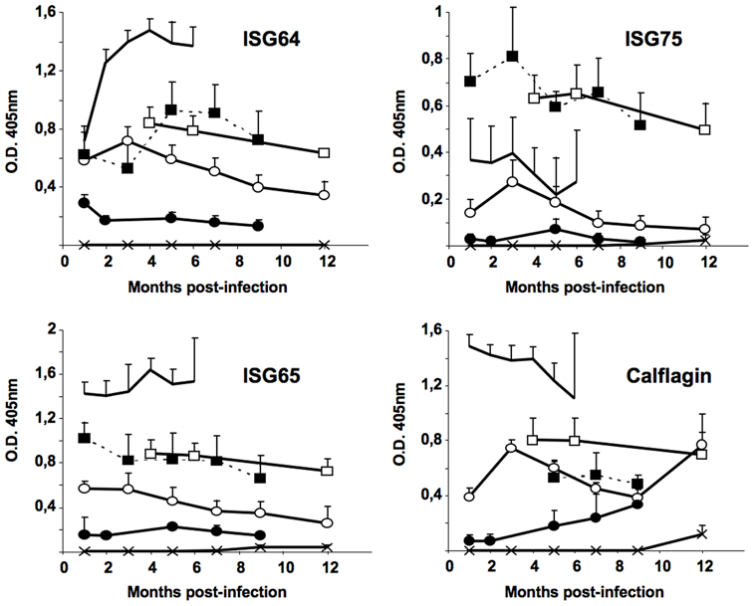
Kinetics of antibody responses to native ISGs and calflagin in BALB/c mice infected with *T. b. gambiense* field isolates. Sera from mice infected with 10^3^ parasites of *Tbg*945b (no symbol), *Tbg*1122c (▪), *Tbg*1122b (□), *Tbg*1135c (x), *Tbg*1135b (○) or with 10^6^ parasites of *Tbg*1135c (•) were collected at different time points PI and tested by ELISA for their reactivity against native ISG64, ISG65, ISG75 and calflagin. Results in *Tbg*1166b (data not shown) and *Tbg*1122b infections were similar. All isolates elicited an antibody response against the recombinant proteins, except the silent *Tbg*1135c (even with a high load of parasites). Each point represents the mean±standard error of 5 mice.

In order to further identify the antigens recognized by immune sera, we compared the Western blot profiles obtained with the sera of infected mice with those obtained with specific antibodies directed against already known immunogenic proteins belonging to the cytoskeleton/membrane fraction: the invariant surface glycoproteins, ISGs (MW 64, 65 or 75 kDa) [45]–[47], the paraflagellar rod protein PFR (MW 70 kDa) [48],[49] and a calflagin isoform (MW 26 kDa) [50] (Fig. 3D). Indeed, ISGs and PFR-specific antibodies recognised proteins from whole cell lysates (Fig. 3D) or the cytoskeleton/membrane fraction (data not shown) with the same molecular weight of those recognised by the sera of infected mice. Furthermore, a calflagin-specific monoclonal antibody (T. Baltz; unpublished results) recognised the three members of the calflagin family (44, 26 and 23 kDa) as did the sera of mice infected with trypanosomes (indicated by arrows in Fig. 3B). These results led us to test these antigens as recombinant proteins by immunoblotting after separation by SDS-PAGE. The recombinant *T. b. gambiense* specific glycoprotein, TgsGP (MW 47 kDa) [42] was included in the test (Fig. 4). All infected mice strongly reacted as soon as one month PI independently of the parasite load inoculated, with the exception of the silent *Tbg*1135c for which a high load was necessary to elicit a significant antibody response. No response was observed in mice infected with a low number of *Tbg*1135c except in one animal (out of 4) for which only PFR was detected. The three antigens ISG65, ISG75 and calflagin were more consistently and strongly recognised, compared to PFR and TgsGP. Interestingly, sera of mice infected with blood isolates always recognised the recombinant proteins earlier and more strongly than the sera of mice infected with culture isolates. To further assess the antibody responses during infection, relative antibody titers were evaluated by ELISA against the native soluble recombinant proteins (ISGs and calflagin). Figure 5 illustrates the results obtained with the sera of mice infected with a low number (1×10^3^) of parasites except for the silent *Tbg*1135c isolate for which the data obtained with a high cell number was included. As observed by Western blotting, all isolates except *Tbg*1135c (even at high load) elicited a significant antibody response against the recombinant proteins. ISG64, ISG65 and calflagin were some of the major immunogenic antigens detected in mice infected with the chronic *Tbg*945 isolate and the sub-chronic *Tbg*1122b, *Tbg*1122c (Fig. 5) and *Tbg*1166c (data not shown) isolates. Low titers of ISG64, ISG65 and calflagin antibodies were detected in mice infected with a low load of *Tbg*1135b.

### Parasite tissue localisation in BALB/c mice infected with *T. b. gambiense* field isolates

**Figure 6:**
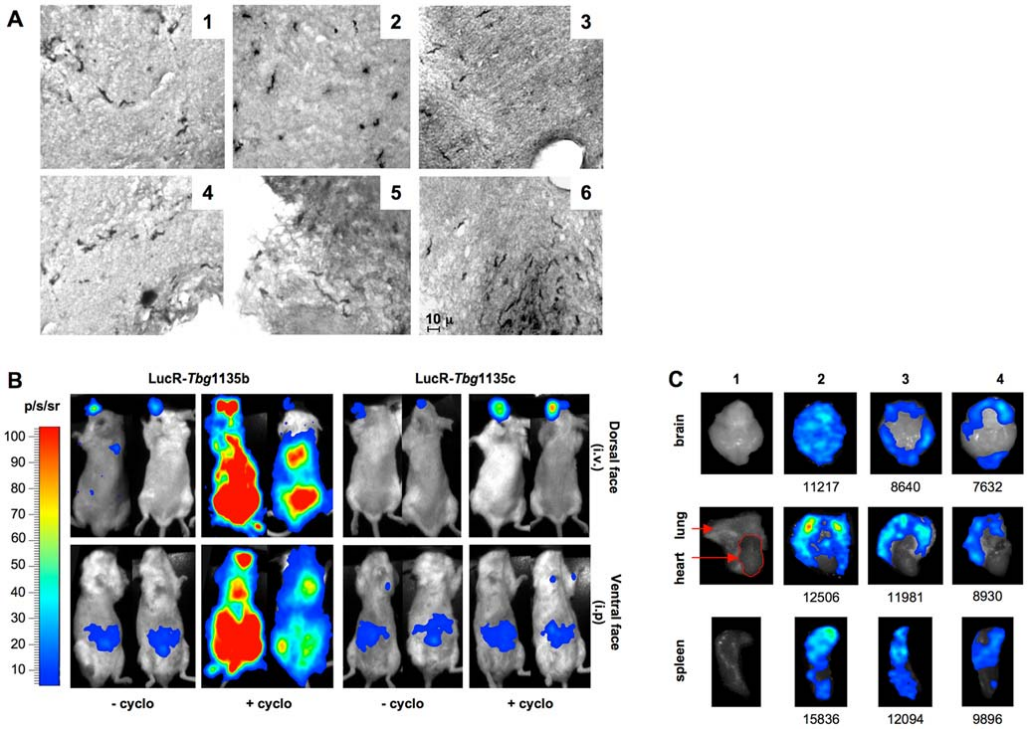
Analysis of *T. b. gambiense* organs and central nervous system invasion in BALB/c-infected mice. A. Immunohistochemical detection of trypanosomes in the brains of mice (n = 2, only results from one mouse are shown) infected for 4 months with 10^6^ parasites of the *Tbg*945b isolate (1, 2, 3) and of paralyzed mice (n = 2) treated with cyclophosphamide before infection with either 5×10^6^ parasites of subchronic *Tbg*1122c or *Tbg*1166c isolates. Paralysis occurred 10 and 6 months PI with *Tbg*1122c and *Tbg*1166c isolates respectively. Only results from *Tbg*1122c are shown (4, 5, 6). A1 and A4 correspond to an olfactory bulb coronal section, A2 to a forebrain section, A3 and A5 to brain stem sections and A6 to a cerebellum section. Whole brain invasion was observed with the chronic isolate at an advanced stage of the disease (4 months PI, death within 6–8 months). Invasion was restricted to the olfactory bulb and the brain stem (including the cerebellum for *Tbg*1122c) in paralyzed mice infected with the sub-chronic isolates after treatment with cyclophosphamide. No invasion was observed in mice (n = 2) infected for 9 months with 5×10^6^ parasites of subchronic *Tbg*1166c isolate (data not shown). B. Spatial distribution of R-Luc activity in animals developing a sub-chronic or a silent infection and treated with or without cyclophosphamide (+/−cyclo). BALB/c mice were either directly infected with 10^6^ LucR-*Tbg*1135b (n = 6) or LucR-*Tbg*1135c (n = 2) or treated 24 h before infection with cyclophosphamide (n = 2). At different time PI, mice were anaesthetized and injected intravenously (i.v., retro-orbital) or intraperitoneally (i.p.) with coelenterazine and BLI signals were recorded in real time with a Biospace Imaging System. The panels show dorsal and ventral images of 2 representative mice infected for 8–11 weeks: LucR-*Tbg*1135b (11 weeks), LucR-*Tbg*1135c (10 weeks), LucR-*Tbg*1135b+cyclo (9 weeks), LucR-*Tbg*1135+cyclo (8 weeks). C. Spatial distribution of R-Luc activity in organs removed from LucR-Tbg1135 infected BALB/c mice. The different organs shown in this figure were isolated from mice: (1) non infected (control) (2) infected for 18 weeks with 10^6^ LucR-*Tbg*1135b, (3) infected for 18 weeks with 10^6^ LucR-*Tbg*1135c, (4) pre-treated with cyclophosphamide and infected for 16 weeks with 10^6^ LucR-*Tbg*1135c. Quantification data of light emission signals for ROI delimitating each organ are given in photons/second/cm^2^/steradian (p/sec/cm^2^/sr).

In order to address CNS invasion in the different models, the presence of parasites in the brain tissues was initially investigated by immunohistochemistry from microtome derived brain sections. In the chronic model (BALB/c mice infected with 1×10^6^ *Tbg*945b parasites, n = 2), parasitic invasion of the brain parenchyma was observed as soon as 4 months PI without any sign of paresis of the mice. Numerous parasites were observed in the olfactory bulb, and in the forebrain where they were widely spread in cerebral cortex, hippocampus and hypothalamus but absent from the myelinated fiber tracts (optic chiasma or corpus callosum). Fewer parasites were present in the brain stem (Fig. 6 A1, A2 and A3). However, no parasites could be detected in the brain of BALB/c mice (n = 2), 9 months after infection with sub-chronic *Tbg*1166c and without clinical signs of disease. In contrast, when mice were cyclophosphamide-immunosuppressed before infection with the sub-chronic isolates, histological examination of the brain of two of the four mice with hind feet paresis (*Tbg*1166c and *Tbg*1122c 6 and 10 months after infection respectively) clearly showed a significant brain invasion by the parasite, restricted to the olfactory bulb and mainly to the brain stem, where they are localised in fiber tracts such as the spinal trigeminal tract (Fig. 6 A4 and A5). In one animal (*Tbg*1122c infected), clusters of parasites could be detected in the cerebellum near a blood vessel (Fig. 6 A6). The limited sensitivity of histological examination due to the fact that only the presence of labelled whole trypanosomes was considered as positive led us to verify the absence of parasites in the brain of mice infected with the sub-chronic isolates (*Tbg*1135b, *Tbg*1122b) by PCR. Nine out of 10 mice tested 5 to 10 months PI, were positive clearly suggesting that brain invasion probably occurs in all models but with differing levels of severity.

The development of an *in vivo* luminescent imaging for *T. b. brucei* using *Renilla* Luciferase-tagged trypanosomes [37] allowed us to apply this method to *T. b. gambiense*. The *Tbg*1135c isolate was transfected with the *Renilla* Luciferase (LucR) vector using the Amaxa Nucleofector system. Stable transfectants (LucR-*Tbg*1135c) were selected, clonally expanded and tested for *in vitro* luciferase activity (807±59 RLU/µg protein) as described in Material and Methods. LucR-*Tbg*1135b parasites were obtained by infecting NOG mice with 10^6^ LucR-*Tbg*1135c and 15–20 days after infection R-Luc activity was measured to assess that they constitutively express R-Luc.

In order to identify the spatio-temporal localisation of parasites in sub-chronic and silent infections, 1×10^6^ trypanosomes derived from LucR-*Tbg*1135b or LucR-*Tbg*1135c isolates were inoculated in BALB/c mice treated with cyclophosphamide, (n = 2 for each isolate) or untreated (n = 6 and n = 2 respectively). The temporal course of invasion and the tissue tropism of the parasite were monitored using real-time *in vivo* bioluminescence imaging (BLI) with coelenterazine as substrate. The BLI signals, either after an i.v. or i.p. injection, of the substrate were analyzed in the untreated mice at different weeks PI. BLI signals were detected as soon as 2 weeks PI in the vicinity of the peritoneum (2 out of 6 mice) and in the front region of the head (4 out of 6 mice) in mice infected with LucR-*Tbg*1135b while no parasite could be detected in the blood. Both mice infected with the silent LucR-*Tbg*1135c gave BLI signals 4 weeks PI. Despite the heterogeneity of the individual values when comparing the data, BLI signals either remained steady or increased slightly during the time course of infection and a BLI signal was detected in all mice 8–13 weeks PI (Fig. 6B). As described previously [51] higher signals were recorded in the head when the substrate was injected i.v rather than i.p. probably due to a better local availability of the substrate. When the dorsal face of the mouse was exposed, the signal was higher in the front region of the head. In cyclophosphamide-treated mice, BLI signals were only recorded long after infection (8 and 9 weeks PI for *Tbg*1135c and *Tbg*1135b respectively) due to the fact that mice become very sensitive to anaesthesia and often die spontaneously. The BLI signals and their increase over time were much higher in the *Tbg*1135b-infected mice than in those infected with *Tbg*1135c (Fig. 6B).

These results prompted us to refine the localisation by recording *ex vivo* BLI on individual organs (brain, lung, spleen, stomach, kidneys, heart, liver and intestines). One animal per group (16–18 weeks PI with *Tbg*1135b, *Tbg*1135c, or *Tbg*1135c after cyclophosphamide treatment) was sacrificed and the dissected organs incubated with coelenterazine and analysed for photon emission. In all animals marked BLI signals (>7 000 photon/s/cm^2^/sr) were observed in the brain, lung and spleen. The other organs displayed no signal or a background signal when properly dissected from the adipose tissue which always gave a positive signal (data not shown). Further histology analyses are needed to characterize the tissue. In the brain, the signal distribution either covered the whole organ (mouse infected with *Tbg*1135b fig. 6C) or was restricted mainly to the olfactory bulb and the cerebellum region (mouse infected with *Tbg*1135c fig. 6C). The distribution was independent of cyclophosphamide treatment.

## Discussion

The successful aim of this study was to provide a murine model based on *T. b. gambiense* isolates to further the understanding of chronic HAT and its epidemiology. So far, most of the host-parasite model systems have been developed with the livestock pathogen *Trypanosoma brucei brucei,* which often induces high parasitaemia that requires treatment with drugs such as suramin or berenil (diminazene aceturate). Since these drugs do not cross the blood-brain barrier (BBB), parasites are cleared from the vascular compartment but not the CNS thereby inducing a chronic infection that could reflect HAT [6], [52]–[54]. As most of the *T. b. gambiense* isolates are not infectious in mice, *T. b. gambiense* models relied on a few rodent adapted isolates producing subacute or chronic infections [16]–[20] characterized by short animal survival time. The major challenge for the development of a long lasting chronic model relied on the adaptation of *T. b. gambiense* directly isolated from the CSF of HAT patients to axenic culture conditions. The presence of fibroblasts as feeder cells and the origin of the sera supplementing the culture medium were the main factors for success in the isolation of 34 (out of 37) field stocks [40]. After rapid adaptation to axenic culture, to avoid growth selection, 3 field isolates were cloned and tested for their infectivity in immunocompetent and immunodeficient mice. These isolates induced infections ranging from chronic to silent in immunocompetent BALB/c mice. In the chronic infection (*Tbg*945), successive waves of parasitaemia were observed and all infected mice died within 8 months. The sub-chronic (*Tbg*1122, *Tbg*1166 and *Tbg*1135b) and silent (*Tbg*1135c) isolates induced longer lasting infections (more than 12 months) without, in most mice, any clinical sign of disease. In addition, parasitaemia was undetectable by microscopy, except for the sub-chronic isolates in which a single low peak of parasitaemia was observed soon after infection. Nevertheless, we clearly provide evidence for the persistence of parasites in all infected mice by PCR and by *in vivo* bioluminescence imaging. Whether the different types of infections are due to host or parasite factors has been addressed by molecular characterisation of the isolates and by analysing the antibody immune responses elicited during the infections. The characterisation of the isolates by micro and mini-satellite analysis showed that all three isolates belonged to the homogeneous group I although some genetic polymorphism was observed despite their common origin from the same endemic area (Daloa, Ivory Coast). These minor genotype differences could explain the differences in infectivity of the isolates. Infection of immunodeficient NOD/SCID mice demonstrated that the innate immune response was not sufficient to contain an infection with *T. b. gambiense* isolates, except for the silent *Tbg*1135c for which the level of parasitaemia was controlled during 1–2 months before fatal parasitaemia involving a new trypanosome variant *Tbg*1135b. Furthermore, parasite host-adaptation occurred, because *Tbg*1135b is more virulent than *Tbg*1135c. These results demonstrate that mice infectivity properties are not only the result of isolate specificities but may change during adaptation to a new host. Table 3 summarizes the different biological properties of the *T. b. gambiense* field isolates.

**Table 3:** Biological properties of type 1 *T. b. gambiense* field isolates.

	*In vitro* culture	Mice infection				
		BALB/c				NOD/SCID
		Virulence survival	Total IgM level	Antibody response	Brain Invasion	Virulence survival
945	−	Chronic<8 months	+	+++	High	Acute
1122, 1166	+	Subchronic>12 months	−	++	Low	Acute
1135c	+	Silent>12 months	−	±	Low	Acute after controlled infection

HAT resulting from *T. b. gambiense* infection and experimental chronic trypanosomiasis induce a non-specific immune response resulting in high blood IgM levels, which is one of the criteria used for diagnosis [44],[55],[56]. In our mice models we clearly demonstrate that the level of macroglobulinemia is linked with the parasite load. Whereas total IgM level increased significantly in the chronic *Tbg*945 infection compared to non-immune mice, sub-chronic or silent isolates did not induce a significant increase of total IgM. Therefore in HAT, absence of macroglobulinemia cannot be considered as a parameter of absence of infection.

Additionally, specific antibody responses were induced in the three infection models as demonstrated by Western blotting against total trypanosome lysates, except in mice infected with a low load of the silent isolate, *Tbg*1135c. We identified several immunogenic antigens belonging to the cytoskeleton/membrane fraction, which are recognised by the infected mice in a similar fashion as the total lysate. By analyzing the banding pattern of the purified recombinant protein strips, ISG65, ISG75 and calflagin always gave a positive reaction. Analyzing the kinetics of antibody responses by ELISA showed that ISG64, ISG65 and calflagin were some of the major invariant antigens which stimulated antibodies in all infections except the silent *Tbg*1135c, even with a high load of parasites. Taken together, specific antibody responses could be clearly detected along the time course of all experimental *T. b. gambiense* infections except when mice were infected with a low load of the silent *Tbg*1135c isolate. These markers may provide new tools for the development of HAT serodiagnosis based on trypanosome invariant antigens. Currently, serological diagnosis of HAT relies on the use of selected highly immunogenic VSGs expressed early in infection by all *T. b. gambiense* isolates (CATT or LATEX/*T. b. gambiense* tests). However, HAT with a parasitaemia that is undetectable by microscopy, as well as the absence of trypanosome-specific antibodies, remains a major concern in the field. Only PCR will be sensitive enough for diagnosis if performed at least twice during the course of infection.

Recently, innovation in imaging technology and the discovery of new bioluminescent markers such as the *Renilla* luciferase facilitated the development of bioluminescent-transfected pathogens thereby allowing the *in vivo* spatio-temporal course of infection to be followed [57]. The development of *in vivo* luminescent imaging for *T. b. brucei* using *Renilla* Luciferase-tagged trypanosomes [37] and the transfection conditions for *T. b. gambiense*, allowed us to obtain stable LucR-transfected trypanosomes. These are LucR-1135b, which behaves as a sub-chronic isolate and *Tbg*1135c, which remains cryptic during the whole course of infection. BLI monitoring of infected BALB/c mice revealed a rapid spread and expansion of parasites (2–4 weeks after inoculation) at two important anatomical sites: the front of the head and the abdomen. We showed that early after infection (16–20 weeks), during the cryptic phase of parasitaemia, trypanosomes are found in a privileged site such as the brain and in other organs such as the spleen and lungs. No specific signal was detected in the heart, liver or kidneys. The maintenance or slight increase over time of the photon emission could only result from active parasite proliferation involving new antigenic variants escaping the immune response of the host but we cannot exclude that parasites may also accumulate in small capillaries (lungs) after proliferation. It is clear that trypanosome proliferation takes place in a limited space where the parasites probably find the proper growth conditions such as endothelial cell attachment [58], a reducing environment [40], nutrients, absence of trypanolytic compounds which might be present in the peripheral blood etc. At that stage of the study it was not possible to precisely localise the parasites within the spleen and lungs. In the brain, however, photon emission was always localised in the olfactory lobes and the cerebellum, the signal was spread over the whole organ in the case of the mouse infected with *Tbg*1135b. The signal distribution reflects both the parasites present in the vascular compartment and those, which could have already infiltrated the brain parenchyma. Nevertheless, the pattern obtained by BLI with the mice infected with *Tbg*1135c after cyclophosphamide treatment is similar to that obtained by immunohistochemistry revealing that parasite brain invasion is mainly restricted to the olfactory bulb, brain stem and cerebellum, a location which might be linked to mouse hind-leg paralysis.

One can speculate that parasites grow attached to specific microvascular endothelial cells [58] present in the brain, spleen or lungs where they undergo antigenic variation and induce variant-specific antibodies. This stimulated immune response may result in trypanolytic activity inducing the release of parasite proteins. In particular, cysteine proteases may be released, which have been shown to induce the activation of brain microvascular endothelial cells allowing the transendothelial migration of *T. b. rhodesiense in vitro* [59],[60]. Furthermore, the continuous lysis of trypanosomes will generate immune complexes, which will elicit a complement-mediated local inflammation establishing the conditions for the parasite to progressively invade the brain through the BBB [60],[61]. Once the parasites have crossed the BBB, they are protected from the immune response and most of the trypanocidal drugs. It is clear from this study that *T. b. gambiense* spreads rapidly (within weeks) to organs such as the brain, lungs and spleen where they multiply and can invade the parenchyma. The severity of the disease and brain invasion clearly depends on the isolate. A *Tbg*945 induced chronic infection, with waves of parasitaemia, will elicit a more severe inflammation in the blood vessels than the sub-chronic or silent isolates, which will result in a more severe invasion of the brain parenchyma as observed by immunohistochemistry and additionally, the rapid death of the animals. Studies defining parasite virulence and host determinants for the disease have been essentially based on *T. b. brucei* mouse infections, in which the course of the disease is rather acute. Furthermore, it has been shown on a human *in vitro* model that *T. b. rhodesiense* is able to cross the BBB more efficiently than *T. b. brucei* [58]. This peculiarity defines *T. b. rhodesiense* as a typical CNS tropic organism whose capacity to invade the brain parenchyma *in vivo* will be highly enhanced depending on the severity of the immune response. Our preliminary results on *T. b. gambiense* brain invasion in mice indicate a major tropism for the olfactory bulb for all isolates, which has not been observed for *T. b. brucei.* The early tropism of the silent *Tbg*1135c isolate and the long lasting, asymptomatic phase in mice are in favour of either, a slow invasion of the organs and/or a slow progression within the organs. Compared to *T. b. brucei* infections, murine infections of *T. b. gambiense* field isolates better mimic the different progressions of gambiense sleeping sickness. In particular, the asymptomatic phase raises the question of “trypano-tolerance” not only in humans but also in animals, which might constitute the natural reservoir for the disease. Indeed, numerous studies suggest that asymptomatic and/or fluctuating carriers with undetectable parasitaemia may occur in the field associated with epidemiological incidences [3],[12],[62],[63]. Furthermore, even if studies have been dedicated to identify markers for the determination of disease stage [64],[65], the duration of the stages, the parasite progression at different stages within the organs, their accessibility to current therapeutic treatments and the outcome of treatment [66],[67] are still elusive. These questions will be addressed [3] by combining different experimental approaches: treatment of infected animals with drugs such as suramin or berenil (diminazene aceturate) which do not cross the BBB and therefore clear the parasites from the vascular compartment and non sedated (to avoid anaesthesia which might kill infected animals) animal BLI analysis, BLI and immunohistochemical analysis of dissected organs, infection of mice with *T. b. gambiense* isolates expressing GFP and optical fluorescence analysis on tissue sections *etc*.

In conclusion, a method has been developed for a reliable adaptation of *T. b. gambiense* isolated from patients to growth in mice. Previous protocols for the adaptation of *T. b. gambiense* to growth in mice were limited. The progression of three different isolates grown in mice were characterised and each produced different outcomes that mirror the different types of human disease: chronic, sub-chronic and silent. The availability of mouse models with a range of disease states will greatly benefit further investigation of disease progression. The murine infections were characterised by measuring parasite growth and the hosts' antibody response. In addition, the distribution of the parasites in the host was determined for one trypanosome isolate by introducing a R-Luc transgene so that it could be visualised in the host. Early in the infection, there was an unexpected tropism not only for the brain but also for other organs such as the spleen and lungs. Using the mouse model we have developed, we can address important questions regarding the molecular mechanisms involved in virulence, sequestration and tropism of *T. b. gambiense* in a more focused manner. This has implications in understanding parasite biology, chemotaxis, blood brain barrier, immune response, pathogenesis and the development of new tools for stage determination during disease progression, and more efficient and less toxic trypanocidal compounds.
