# Correction: Murine Models for *Trypanosoma brucei gambiense* Disease Progression—From Silent to Chronic Infections and Early Brain Tropism

**DOI:** 10.1371/journal.pntd.0004645

**Published:** 2016-04-19

**Authors:** Christiane Giroud, Florence Ottones, Virginie Coustou, Denis Dacheux, Nicolas Biteau, Benjamin Miezan, Nick Van Reet, Mark Carrington, Felix Doua, Théo Baltz

Fig 4 is incorrect. The two Western blot strips for the high load of *Tbg*945b for 3 and 5 months and the two Western blot strips for the low load of *Tbg*1135c for 1 and 7 months were duplicated. Please see the corrected figure below.

**Fig 4:**
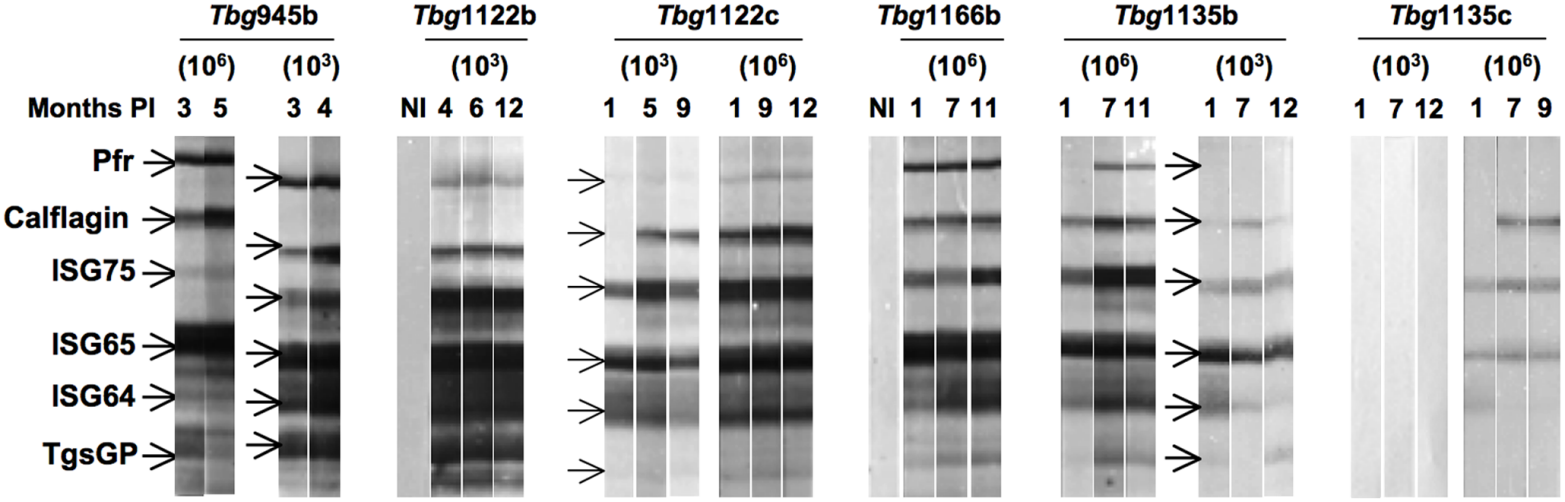
Reactivity patterns of immunoreactive invariant trypanosome proteins during BALB/c mice infections. Four mice were infected with either a low (103) or a high (106) load of Tbg945b, Tbg1122b, Tbg1166b, Tbg1135b or Tbg1135c isolates and their sera collected at different time points were tested by Western blotting (1/100 dilution) against a strip loaded with recombinant protein: 0.5 μg PFR and ISG75, 1 μg ISG65, ISG64 and TgsGP and 2 μg calflagin. The data are representative of one immunoblot out of 4 mice tested. NI represents the control sera before infection.

## Supporting Information

S1 Fig Raw images of the Western blot strips with their corresponding experiments and the final cropped image included in the corrected Fig 4. The raw uncropped image was taken with a Panasonic DMC-FZ50 digital camera. The relationship between the strip numbers, the experiments and the original pictures is listed in the table.
The two Western blot strips for the high load of Tbg945b for 3 and 5 months correspond to strip 42 and 43 respectively. The two Western blot strips for the *Tbg*1135c low load for 1 and 7 months correspond to the strip 14 and 15 respectively. The corrections of Fig 4 were made with the cropped images of the strips N° 14, 15, 42 and 43 issued from the original colour images taken with the digital camera
(PDF)
