# Supplementary material for: Correction: Murine Models for Trypanosoma brucei gambiense Disease Progression—From Silent to Chronic Infections and Early Brain Tropism
Source: PLoS Negl Trop Dis. 2016 Apr 19;10(4):e0004645. doi: 10.1371/journal.pntd.0004645 (PMC4836668; doi:10.1371/journal.pntd.0004645)
Supplement: S1 Fig — The raw uncropped image was taken with a Panasonic DMC-FZ50 digital camera. The relationship between the strip numbers, the experiments and the original pictures is listed in the table. The two Western blot strips for the high load of Tbg945b for 3 and 5 months correspond to strip 42 and 43 respectively. The two Western blot strips for the Tbg1135c low load for 1 and 7 months correspond to the strip 14 and 15 respectively. The corrections of Fig 4 were made with the cropped images of the strips N° 14, 15, 42 and 43 issued from the original colour images taken with the digital camera (PDF) [file pntd.0004645.s001.pdf]

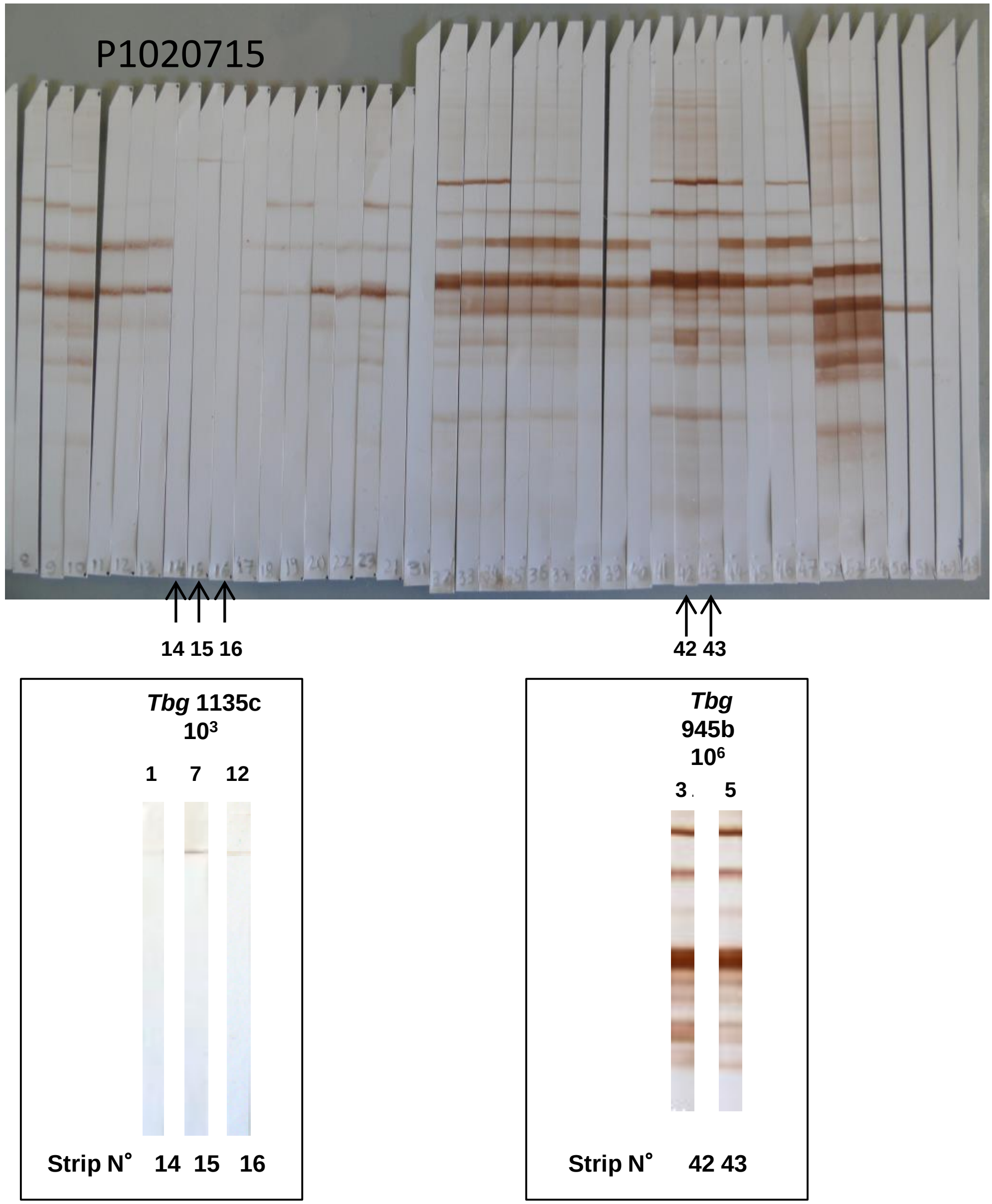

Strip N°	Experiment	Parasite load	Time point (months)	Files
14	*Tbg*1135c	1E+04	1	Strip Flo.1sc.jpg, 7-23.jpg, P1020715
15	*Tbg*1135c	1E+04	7	Strip Flo.1sc.jpg, 7-23.jpg, P1020715
16	*Tbg*1135c	1E+04	12	Strip Flo.1sc.jpg, 7-23.jpg, P1020715
17	*Tbg*1135c	1E+07	1	Strip Flo.1sc.jpg, 7-23.jpg, P1020715
18	*Tbg*1135c	1E+07	7	Strip Flo.1sc.jpg, 7-23.jpg, P1020715
19	*Tbg*1135c	1E+07	9	Strip Flo.1sc.jpg, 7-23.jpg, P1020715
20	*Tbg*1135b	1E+04	1	Strip Flo.1sc.jpg, 7-23.jpg, P1020715
21	*Tbg*1135b	1E+04	7	Strip Flo.1sc.jpg, 7-23.jpg, P1020715
22	*Tbg*1135b	1E+04	12	Strip Flo.1sc.jpg, 7-23.jpg, P1020715
31	*Tbg*1166b	1E+07	NI	Strip Flo.1sc.jpg, P1020715, P1020695, P1020711
32	*Tbg*1166b	1E+07	1	Strip Flo.1sc.jpg, P1020715, P1020695, P1020711
33	*Tbg*1166b	1E+07	7	Strip Flo.1sc.jpg, P1020715, P1020695, P1020711
34	*Tbg*1166b	1E+07	11	Strip Flo.1sc.jpg, P1020715, P1020695, P1020711
35	*Tbg*1122c	1E+07	1	Strip Flo.1sc.jpg, P1020715, P1020695, P1020711
36	*Tbg*1122c	1E+07	9	Strip Flo.1sc.jpg, P1020715, P1020695, P1020711
37	*Tbg*1122c	1E+07	12	Strip Flo.1sc.jpg, P1020715, P1020695, P1020711
38	*Tbg*1122c	1E+04	1	Strip Flo.1sc.jpg, P1020715, P1020695, P1020711
39	*Tbg*1122c	1E+04	5	Strip Flo.1sc.jpg, P1020715, P1020695, P1020711
40	*Tbg*1122c	1E+04	9	Strip Flo.1sc.jpg, P1020715, P1020695, P1020711
42	*Tbg*945b	1E+07	3	P1020715, P1020695, P1020711
43	*Tbg*945b	1E+07	5	Strip Flo.1sc.jpg, P1020715, P1020695, P1020711
45	*Tbg*1135b	1E+07	1	Strip Flo.1sc.jpg, P1020715, P1020695, P1020711
46	*Tbg*1135b	1E+07	7	Strip Flo.1sc.jpg, P1020715, P1020695, P1020711
47	*Tbg*1135b	1E+07	11	Strip Flo.1sc.jpg, P1020715, P1020695, P1020711
49	*Tbg*1122b	1E+04	NI	Strip Flo.1sc.jpg, P1020715, P1020695, P1020711
52	*Tbg*1122b	1E+04	4	Strip Flo.1sc.jpg, P1020715, P1020695, P1020711
53	*Tbg*1122b	1E+04	6	Strip Flo.1sc.jpg, P1020715, P1020695, P1020711
54	*Tbg*1122b	1E+04	12	Strip Flo.1sc.jpg, P1020715, P1020695, P1020711
55	*Tbg*945b	1E+04	3	Strip Flo.1sc.jpg, P1020715, P1020695, P1020711
56	*Tbg*945b	1E+04	4	Strip Flo.1sc.jpg, P1020715, P1020695, P1020711

Table: relationship between strip numbers, experiments and raw image files.
